# The role of bHLH-PAS transcription factors in endoplasmic reticulum stress

**DOI:** 10.3389/fmolb.2025.1730502

**Published:** 2025-12-12

**Authors:** Izabela Krauze, Małgorzata Krzystek-Korpacka, Kamila Maciejewska, Beata Greb-Markiewicz

**Affiliations:** 1 Department of Biochemistry, Molecular Biology and Biotechnology, Wroclaw University of Science and Technology, Wrocław, Poland; 2 Department of Biochemistry and Immunochemistry, Wroclaw Medical University, Wrocław, Poland

**Keywords:** bHLH-PAS domain proteins, transcriptional factors, endoplasmic reticulum stress, cellularstress, therapeutic targets

## Abstract

The bHLH-PAS protein family consists of transcription factors that are involved in the regulation of key physiological processes such as the response to hypoxia, circadian rhythms, the detoxification of xenobiotics, and metabolic homeostasis. These proteins act as environmental sensors, integrating diverse signals into transcriptional responses. In recent years, increasing attention has been paid to their role in regulating endoplasmic reticulum stress (ER stress), which is an adaptive cellular response to disturbances in protein-folding. Prolonged or severe ER stress can activate the unfolded protein response (UPR) and apoptotic pathways, contributing to the development of numerous disorders, including neurodegenerative, cancerous, and inflammatory diseases. This review focuses on the functions of bHLH-PAS proteins, such as AHR, HIF, SIM, NPAS1-4, and CLOCK, with particular emphasis on their potential role in modulating ER stress. Molecular mechanisms through which these proteins regulate responses to hypoxia and other cellular stressors are also discussed, with a focus on their importance in maintaining homeostasis and their potential as therapeutic targets.

## 1 Introduction

One of the key cellular mechanisms in response to disrupted homeostasis is the endoplasmic reticulum (ER) stress response (Chen et al., 2023). The ER is responsible for the proper folding of proteins, their post-translational modifications, and transport. Under conditions of ER stress, such as protein overproduction, hypoxia, redox imbalance, or nutrient deprivation, misfolded or unfolded proteins begin to accumulate. In response, the cell activates a protective mechanism known as the Unfolded Protein Response (UPR), which aims to restore balance by temporarily inhibiting translation, increasing the expression of molecular chaperones, and activating pathways that degrade aberrant proteins. The UPR is essential for cell survival under stress conditions, enabling adaptation to environmental changes and maintaining the integrity of intracellular structures (Read and Schröder, 2021). However, prolonged or excessive ER stress can lead to the activation of pro-apoptotic pathways, including the transcription factor CHOP, the kinase PERK, and caspases, which can ultimately result in cell death (Liu et al., 2024). Chronic ER stress plays a significant role in the pathogenesis of various diseases, including diabetes, neurodegenerative disorders, cancer, and inflammatory diseases (Oakes and Papa, 2015). Studies indicate that proteins from the basic helix-loop-helix-PER-ARNT-SIM (bHLH-PAS) family may directly or indirectly influence the course of ER stress and the UPR. This is due, among other factors, to their role in regulating numerous cellular and physiological processes such as development, stress response, circadian rhythms, the response to hypoxia, and the detoxification of xenobiotics (Edwards and Gorelick, 2022). The defining feature of bHLH-PAS family members is the presence of conserved bHLH and PAS domains. These domains enable DNA binding and the formation of heterodimers, which are essential for transcriptional activation, and functioning as environmental sensors that integrate physiological signals with transcriptional responses (Kolonko and Greb-Markiewicz, 2019). Despite an increasing number of reports in the literature, the mechanisms linking bHLH-PAS proteins and ER stress remain incompletely understood. This work aims to provide a comprehensive review and summary of the current state of knowledge regarding these interactions, with a particular focus on their potential role in maintaining cellular homeostasis. Considering the diverse regulatory functions of bHLH-PAS proteins, they appear to be an important, yet insufficiently explored, component of the signaling network responsible for cellular adaptation to stress conditions. This network is critical in preventing the development of various metabolic and neurodegenerative diseases, as well as cancers.

### 1.1 General characteristics of bHLH-PAS proteins

**FIGURE 1:**
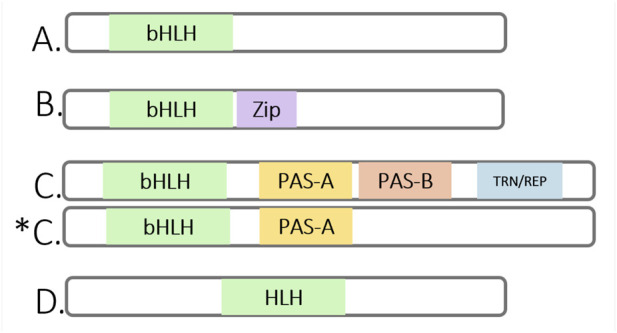
Schematic representation of the bHLH protein family, including representatives of different classes and the domain structures of selected members. **(A)** Proteins containing only the bHLH (basic Helix-Loop-Helix) domain; **(B)** bHLH proteins with an additional Zip motif (leucine zipper) involved in dimerization; **(C)** bHLH proteins with a PAS domain, containing PAS-A and PAS-B repeats – where PAS-A is responsible for specific binding to the dimerization partner and stabilization of the DNA complex, while PAS-B functions as a sensory domain responsible for recognizing small molecules (e.g.,: ligands, oxygen, or xenobiotics). These proteins often also contain domains responsible for transcriptional activation or repression (TRN/REP); * **(C)** bHLH proteins containing only one PAS-A repeat, such as AhRR (Aryl Hydrocarbon Receptor Repressor); **(D)** bHLH proteins lacking the *basic* region, and therefore unable to bind DNA – examples include proteins from the Id family (Inhibitor of DNA binding), which perform regulatory functions by sequestering active bHLH proteins (Kewley et al., 2004; Jones, 2004); (Figure prepared with BioRender).

Based on the phylogenetic classification dividing bHLH family of transcription factors in four monophyletic groups of proteins containing the bHLH domain, bHLH-PAS proteins characterized by the presence of additional PAS domain belong to the C group of bHLH family of transcription factors (Figure 1). Other groups are known as group A, B and D. Group A comprises both tissue-specific and ubiquitously expressed proteins that contain only the bHLH domain and act as key transcriptional regulators by binding to E-box sequences in target gene promoters. Group B includes functionally diverse proteins that possess an additional leucine zipper domain, which facilitates homo- and heterodimerization with other members of the bHLH family. Group D comprises proteins that lack the basic DNA-binding region and therefore cannot bind DNA. These proteins act as antagonists of group A proteins by modulating their function through the formation of inactive dimeric complexes. All groups are derived from a common ancestor and include all its descendants (Atchley and Fitch, 1997; Ledent and Vervoort, 2001; Skinner et al., 2010).

The bHLH domain was first identified in the murine transcription factors E12 and E47 (Murre et al., 1989). During subsequent research, the domain was found in many other proteins in animals, plants, and fungi. To date, approximately 125 human bHLH proteins have been identified (Ledent et al., 2002). The bHLH domain consists of around 60 amino acid residues and is a highly conserved structure located in the N-terminal region. It comprises two functionally distinct parts: a basic region characterized by a sequence of positively charged (basic) amino acids such as lysine and arginine, that is responsible for DNA binding and a C-terminal HLH region which is involved in protein dimerization (Kolonko and Greb-Markiewicz, 2019; Li et al., 2006). The bHLH domain is responsible for recognizing specific DNA sequences, such as the E-box motif (CANNTG), as well as interacting with other proteins toenable dimerization and regulation of transcription (Ledent and Vervoort, 2001). It promotes dimerization and the formation of homodimeric and heterodimeric complexes between members of the bHLH protein family.

The PAS domain has been identified in wide range of proteins from various organisms, including bacteria, fungi, invertebrates, and mammals. The term PAS is derived from the first letters of the names of the three proteins in which this domain was initially identified: Per (Period), Arnt (Aryl hydrocarbon receptor nuclear translocator), and Sim (Single-minded). The PAS domain is usually located in the central region of a protein (Crews and Fan, 1999). A total of 34 proteins containing the PAS domain have been identified in the human genome. These proteins can be divided into four families: KCNH (potassium ion channels), PDE8 (Phosphodiesterases), PASK (Serine/Threonine Kinases), and bHLH-PAS (Xing et al., 2023). The PAS domain consists most often of two structurally conserved regions, PAS-A and PAS-B, each of which comprises approximately 130 amino acid residues. PAS-A repeat plays a crucial role in preventing dimerization with bHLH proteins that lack a PAS domain. The PAS domain typically consists of two structurally conserved regions, PAS-A and PAS-B, each comprising approximately 130 amino acid residues. It is also frequently referred to as the PAC domain (PAS-associated C-terminal domain) (Pont et al., 1997). The PAS-A repeat plays a crucial role in preventing dimerization with bHLH proteins that lack a PAS domain and helps to specify DNA binding by restricting off-target interactions with DNA sequences that differ from the classical E-box motif, which is recognized by most members of the bHLH protein family. In contrast, the PAS-B region is essential for signal transduction by sensing environmental signals and/or binding small molecules, as well as interacting with sensory proteins that transmit this information (Kewley et al., 2004; Taylor and Zhulin, 1999).

**FIGURE 2:**
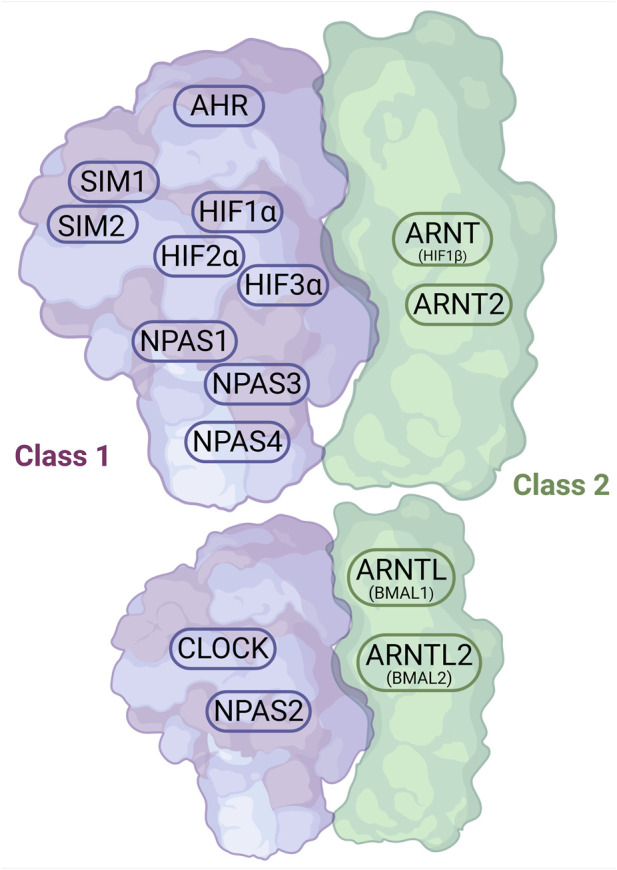
The family of bHLH-PAS proteins – division into two clasess based on the dimerization pattern (Figure prepared with BioRender).

Currently, there are 19 known human proteins belonging to the bHLH-PAS family. All of these proteins contain a conserved N-terminal bHLH domain, followed by a PAS domain. Their C-terminal regions are highly variable and include domains responsible for regulating transcription, either through activation or repression (Kewley et al., 2004). Some of these transcription factors contain only a single PAS repeat—most commonly PAS-A—whereas the majority possess both PAS-A and PAS-B repeats, with the former typically involved in protein–protein interactions and the latter often serving a sensory function (Edwards and Gorelick, 2022). Based on their dimerization pattern, bHLH-PAS proteins are divided into two classes (Figure 2) (Fribourgh and Partch, 2017). Class I proteins form dimers with Class II proteins, as they are unable to homodimerize, whereas Class II proteins can form both homo- and heterodimers. Class I proteins include: AHR (Aryl Hydrocarbon Receptor), HIF1α (Hypoxia-Inducible Factor 1-alpha), HIF2α (Hypoxia-Inducible Factor 2-alpha (also known as EPAS1 – Endothelial PAS Domain Protein 1), HIF3α (Hypoxia-Inducible Factor 3-alpha), SIM1 (Single-minded Homolog 1), SIM2 (Single-minded Homolog 2), NPAS1 (Neuronal PAS Domain-containing Protein 1), NPAS2 (Neuronal PAS Domain-containing Protein 2), NPAS4 (Neuronal PAS Domain-containing Protein 4), and CLOCK (Circadian Locomotor Output Cycles Kaput). Class II proteins include: ARNT (Aryl Hydrocarbon Receptor Nuclear Translocator, HIF1β, Hypoxia-Inducible Factor 1-beta), ARNT2 (Aryl Hydrocarbon Receptor Nuclear Translocator 2), ARNTL (Aryl Hydrocarbon Receptor Nuclear Translocator-Like, BMAL1, Brain and Muscle ARNT-Like Protein 1), and ARNTL2 (Aryl Hydrocarbon Receptor Nuclear Translocator-Like 2, BMAL2, Brain and Muscle ARNT-Like Protein 2) (Edwards and Gorelick, 2022).

### 1.2 Endoplasmic reticulum stress

The endoplasmic reticulum is a membrane bound organelle of eukaryotic cells. It is characterized by an extensive network of biological membranes that form flattened cisternae and branched tubules (Almanza et al., 2019). This network spans nearly the entire cellular space and is continuous with the outer membrane of the nuclear envelope. There are two main types of endoplasmic reticulum, each with different functions: rough endoplasmic reticulum (RER), which contains ribosomes, and smooth endoplasmic reticulum (SER), which lacks ribosomes (Carlson, 2019). The main function of the RER is synthesizing proteins destined for export. This organelle is particularly well developed in cells specialized in intensive protein production, such as the secretory cells of the pancreas. In contrast, the SER is involved in lipid synthesis, including steroid hormones, which is reflected in its extensive development in adrenal cortex cells. A specialized type of smooth ER found in muscle cells is the sarcoplasmic reticulum, which primarily stores Ca^2+^ (Meldolesi et al., 1998; Rossi et al., 2008). Newly synthesized proteins, produced on polyribosomes attached to the RER membrane, are directed into its lumen or membrane, where they undergo post-translational modifications such as signal peptide cleavage, glycosylation, and disulfide bond formation (Ellgaard et al., 2016). Disruptions to protein folding or calcium homeostasis in the ER lead to the accumulation of misfolded polypeptides. This process is regulated by chaperone proteins, such as calnexin and calreticulin; heat shock proteins such as GRP94; and enzymes, such as protein disulfide isomerase (PDI) and peptidyl-prolyl isomerase (PPI) (Fink, 1999; Hebert et al., 1995). Excessive accumulation of misfolded proteins leads to ER stress, which triggers the UPR mechanism aimed at restoring homeostasis within the ER - UPR.

The UPR is initiated by the activation of three main ER transmembrane proteins: PERK (protein kinase R (PKR)-like endoplasmic reticulum kinase), IRE1 (inositol-requiring enzyme 1), and ATF6 (activating transcription factor 6) (Schindler and Schekman, 2009; Harding et al., 1999; Lin et al., 1979). The activation of these factors initiate several cellular responses, including transient attenuation of protein translation, induction of transcription leading to increased expression of molecular chaperones, and activation of the ER-associated degradation (ERAD) pathway (Adachi et al., 2008; Kondratyev et al., 2007; Korennykh et al., 2009). ERAD degrades misfolded proteins. This process involves the selective retro-translocation of proteins from the ER lumen and membrane into the cytosol, where they are subsequently degraded by cytosolic proteasomes. Cytosolic chaperones assist in the delivery of these substrates to the proteasomes. Most proteins targeted for proteasomal degradation undergo ubiquitination (Bays et al., 2001). When the accumulation of misfolded proteins becomes overwhelming and degradation mechanisms are no longer sufficient to restore homeostasis, the apoptotic cell death pathway is triggered (Read and Schröder, 2021; Kim et al., 2022; Lindner et al., 2020).

**FIGURE 3:**
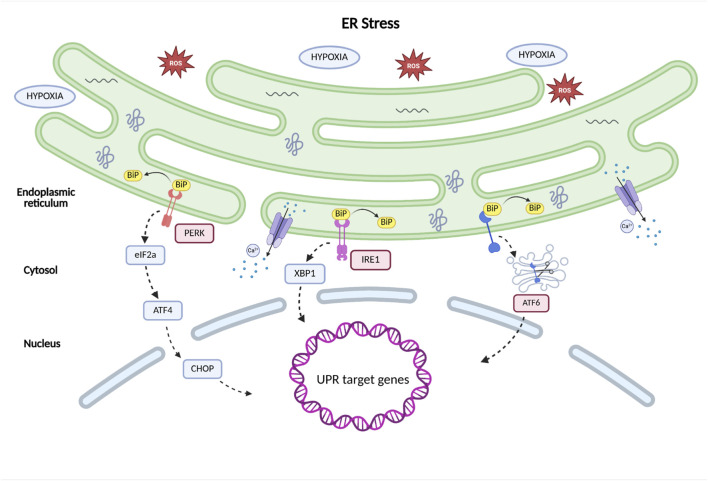
The pathway of the UPR. The activation of the UPR in the endoplasmic reticulum is initiated under conditions of cellular stress, such as hypoxia, oxidative stress, or disturbances in calcium homeostasis. The accumulation of misfolded proteins within the ER leads to the dissociation of the chaperone BiP from the three primary sensors: PERK, IRE1, and ATF6, resulting in their activation. Once activated, these sensors trigger a signaling cascade that induces the expression of UPR target genes, whose function is to restore homeostasis within the endoplasmic reticulum (Figure prepared with BioRender).

The proteins that activate the UPR mechanism are anchored in the ER membrane. These proteins consist of three distinct domains, each of which play a critical role in initiating the response: a luminal domain, responsible for sensing misfolded proteins, a transmembrane domain, which anchors the protein and transmits the signal from the luminal side to the cytosolic side, and a cytoplasmic domain, which mediates downstream signaling and activates genes involved in the ER stress responses. UPR activation also involves the binding immunoglobulin protein (BiP), a molecular chaperone belonging to the heat shock protein 70 (HSP70) family. BIP plays a key role in sensing unfolded proteins and regulating the activation of ER stress sensors (Kopp et al., 2019). In the absence of ER stress, the stress sensors PERK, IRE1, and ATF6 are bound to BiP within the ER lumen. This prevents the activation of the UPR signaling pathway. Upon the accumulation of misfolded proteins and induction of ER stress, BiP dissociates from these sensors, thereby enabling their activation through homodimerization, autophosphorylation, or proteolytic cleavage. This initiates the UPR signaling cascade mediated by PERK, IRE1, and ATF6 (Figure 3) (Korennykh et al., 2009; Tran et al., 1979).

Various factors are known to contribute to ER stress. The primary causes include disruptions to cellular homeostasis resulting from hypoxia and oxidative stress. Hypoxia, which is defined as a reduced oxygen levels in tissues, plays a crucial role in human embryonic development. Under conditions of physiological hypoxia, characterized by low oxygen concentration in the intrauterine environment, transcription factors involved in key developmental processes such as angiogenesis, organogenesis, and the regulation of cellular metabolism are activated. However, scientific literature more frequently focuses on pathological hypoxia, which is associated with various disorders, including ischemia, cardiovascular diseases, stroke, neurodegenerative disorders, chronic inflammation, obesity, and cancer (Ziello et al., 2007).

Hypoxia is one of the main factors that activate the member of bHLH-PAS family - Hypoxia-inducible factor 1 alpha (HIF-1α, which plays a key role in regulating the expression of genes involved in cellular adaptation to metabolic stress. This regulation enables cells to survive under unfavorable environmental conditions characterized by reduced oxygen availability (Chee et al., 2023). A similar relationship involving HIF-1α can also be observed in the context of oxidative stress, which serves as a crucial link connecting regulatory mechanisms mediated by bHLH-PAS family proteins and the UPR pathway. Another bHLH-PAS protein that plays a pivotal role in the cellular response to oxidative stress by activating signaling pathways that promote cell survival and adaptation, is the AHR (Gabriely et al., 2017). Another noteworthy member of the bHLH-PAS family is NPAS4, whose expression is induced by classical endoplasmic reticulum stressors. NPAS4 may play a protective role in preventing dysfunction and cell death of pancreatic β-cells under conditions of ER stress (Sabatini et al., 2013). The following chapter presents a literature review summarizing the current knowledge on the role of individual members of the bHLH-PAS family in regulating the endoplasmic reticulum stress response, as well as their potential significance in pathological processes, including cancer and metabolic diseases.

## 2 bHLH-PAS proteins and ER stress

### 2.1 HIFs - Hypoxia inducible factors

Hypoxia, defined as a reduced oxygen level in tissues, plays a crucial role in human embryonic development. Under conditions of physiological hypoxia, characterized by low oxygen concentrations in the intrauterine environment, transcription factors involved in key developmental processes—such as angiogenesis, organogenesis, and the regulation of cellular metabolism—are activated. However, scientific literature more frequently focuses on pathological hypoxia, which accompanies various disorders including ischemia, cardiovascular diseases, stroke, neurodegenerative diseases, chronic inflammation, obesity, and cancer (Ziello et al., 2007). Hypoxia-inducible factors (HIFs) play a key role in the regulation of oxygen homeostasis. HIFs control the transcription of genes involved in adaptive mechanisms that enable the maintenance of adequate oxygen levels during conditions of oxygen deficiency (Jiang et al., 1997). HIF is a heterodimeric transcription factor composed of two subunits: α and β. In humans, three paralogs of the α subunit have been identified (HIF-1α, HIF-2α/EPAS1, HIF-3α), as well as two paralogs of the β subunit (HIF-1β/ARNT and HIF-2β/ARNT2) (Lee et al., 2004). The β subunit (ARNT) is constitutively expressed, and its levels are independent of oxygen availability. In contrast, the α subunit is regulated in an oxygen-dependent manner. Under normoxic conditions, the α subunit is rapidly degraded by the proteasome. During hypoxia, the α subunit is stabilized, allowing its translocation into the nucleus, where it forms a heterodimer with the β subunit (ARNT). The resulting complex binds to hypoxia response elements (HREs) in the promoters of target genes, initiating the transcription of genes involved in cellular adaptation to oxygen deprivation (Huang et al., 1998; Wang et al., 1995). This cytoprotective function is exhibited during the early stages of hypoxia, whereas prolonged hypoxia may lead to cell death.

**FIGURE 4:**
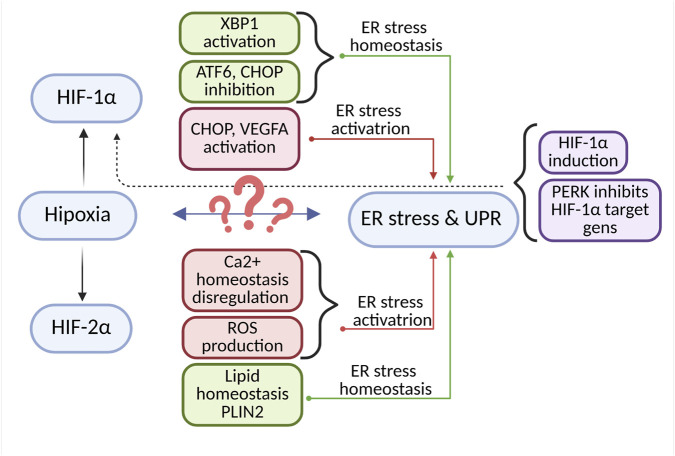
Interplay Between HIF Signaling and ER Stress/UPR. A complex, bidirectional relationship exists between hypoxia, the transcription factors HIF-1α and HIF-2α, and ER stress along with the UPR. Both HIF-1α and HIF-2α may contribute to maintaining ER homeostasis through activation of XBP1, regulation of the ATF6 pathway, inhibition of the pro-apoptotic factor CHOP, and modulation of lipid metabolism (e.g., via PLIN2). Conversely, HIFs can also induce ER stress and activate the UPR by increasing the expression of CHOP and VEGFA, enhancing ROS production, and disrupting calcium homeostasis. In turn, intensified ER stress and UPR may promote the expression of HIF-1α. The diagram was created based on the description provided in Section 2.1. (Figure prepared with BioRender).

Hypoxia influences the activation of ER stress; however, this process does not necessarily depend directly on the HIF transcription factor. Oxygen deprivation induces ER stress through various mechanisms, including the accumulation of ROS and the activation of oxidative stress, disturbances in calcium homeostasis, direct activation of the PERK kinase, as well as improper protein folding leading to their accumulation in the ER lumen (Guan et al., 2024; Zhou et al., 2022). Although HIF is not the primary initiator of ER stress or the UPR, studies suggest its direct or indirect involvement in regulating these processes. HIF-1α can inhibit ATF6 and CHOP, thereby reducing ER stress. It has been observed that silencing of HIF-1α increases the levels of homologous C/EBP protein (CHOP), PERK, and ATF6. Conversely, stabilization of HIF-1α decreases the expression of ER stress markers such as *Ddit3, Hspa5, Atf6*, and *Eif2a*. Chemical inducers of ER stress, such as thapsigargin and tunicamycin, have been shown to induce HIF-1α activity while simultaneously promoting the UPR (Novais et al., 2021). Studies conducted on primary alveolar epithelial cells (AEC) have demonstrated that hypoxia activates UPR pathways, leading to increased expression of CHOP and apoptosis. A concurrent upregulation of HIF-1α and CHOP (a UPR effector) was observed. ER stress inhibitors, as well as inhibition or silencing of HIF-1α, prevented CHOP expression and apoptosis. Furthermore, overexpression of HIF-1α under normoxic conditions enhanced the activity of UPR-associated transcription factors and increased CHOP expression. These findings suggest that HIF-1α may play a key role in inducing ER stress and apoptosis through the regulation of CHOP (Figure 4) (Delbrel et al., 2018). The study by Ameri et al., 2004, revealed that the induction of ATF4 expression (a key component of the UPR) under anoxic conditions in cancer cells is independent of HIF-1α (Ameri et al., 2004). Interactions between HIF-1α and ER stress represent an intriguing example of the cooperation between cellular stress response mechanisms. In breast cancer cell lines, heterodimerization and colocalization of these two proteins have been observed, illustrating the interplay between hypoxia and the UPR. Analysis of samples from patients with triple-negative breast cancer (TNBC) revealed a specific gene expression signature of XBP1, which strongly correlated with HIF-1α expression signatures and was associated with poor prognosis. It was also noted that hypoxia and HIF-1α can induce the expression and splicing of XBP1, leading to an increased level of this protein. Silencing of XBP1 inhibited tumor cell proliferation, metastasis, and enhanced sensitivity to hypoxia-induced apoptosis. XBP1 and HIF-1α interact to enhance the expression of HIF target genes (Chen et al., 2014; Pereira et al., 2014). These findings suggest that HIF-1α plays a significant role in the induction of ER stress and the UPR, which may influence tumor development (Figure 4). It is important to emphasize that cancer cells often develop mechanisms to evade the UPR under severe stress conditions, thereby preventing the activation of apoptotic pathways. Such adaptations contribute to disease progression and worsen the prognosis for patients (Madden et al., 2019). HIF-1α can also activate the UPR through the induction of VEGF expression, which is one of the main target genes of HIF. HIF-1α may stimulate VEGFR receptors, thereby activating phospholipase C, leading to the release of inositol trisphosphate (IP3)-dependent calcium and initiation of the UPR (Karali et al., 2014; Forsythe et al., 1996). Interestingly, it appears that not only can HIF influence ER stress and the unfolded protein response (UPR), but the relationship can also be reciprocal. Studies by Ivanova et al., 2018, indicate that under moderate hypoxia, ER stress reduces the levels and activity of HIF-1α through a PERK-dependent mechanism (Figure 4). It was observed that activation of the PERK pathway suppresses the induction of HIF target genes. Furthermore, UPR activation does not promote the accumulation of HIF-1α but rather inhibits it, suggesting the necessity for alternative signaling pathways to maintain HIF-1α stability. In contrast, hypoxia-dependent accumulation of HIF-2α is not associated with the UPR, indicating specific translational control of HIF-1α mRNA (Ivanova et al., 2018). Activation of ER stress and the UPR can lead to the degradation of HIF-1α mRNA and a reduction in its expression. In studies conducted on a rat neuronal model, prolonged chemical hypoxia induced by CoCl_2_ activated ER stress, which subsequently resulted in the degradation of HIF-1α mRNA and decreased levels of the protein (Lõpez-Hernández et al., 2015). It is also worth paying attention to the HIF-2α isoform. Evidence suggests that its low expression promotes the production of reactive oxygen species (ROS), which in turn activates ER stress and ultimately leads to the death of hematopoietic stem and progenitor cells (HSPCs) (Rouault-Pierre et al., 2013). An indirect effect of HIF-2α on ER stress has also been observed in clear cell renal cell carcinoma (ccRCC) cells. PLIN2 (Perilipin 2) is a lipid droplet-associated protein that plays a key role in lipid storage and regulation of lipid metabolism by coating lipid droplets and protecting them from lipolysis. Proper functioning of PLIN2 is essential for maintaining lipid homeostasis in cells. Studies revealed that the functioning of the HIF-2α–PLIN2 axis, and thus lipid storage in cancer cells, is required for maintaining ER homeostasis. Inhibition of this axis by targeting HIF-2α induced ER stress (Figure 4) (Qiu et al., 2016).

### 2.2 Single minded protein (SIM)

Another member of the bHLH-PAS family is the transcription factor SIM. It is a constitutively nuclear protein present in two isoforms, SIM1 and SIM2. SIM1 activates the expression of target genes, while SIM2 represses it. Removal of their C-terminal domains abolishes this difference, resulting in similar activity for both proteins. mSIM-1 and mSIM-2 are mammalian homologs of Sim in *Drosophila*. mSIM-1 forms a heterodimer with ARNT, activating transcription through the CNS midline enhancer (CME), whereas mSIM-2 acts antagonistically by inhibiting ARNT-dependent activation and competing for ARNT binding (Moffett and Pelletier, 2000). Studies on the function of SIM in human models are severely limited, with most research focusing on mouse models. SIM plays a key role in the development and function of paraventricular hypothalamic neurons. The SIM gene acts as a genetic switch, determining the development of a specific population of neuroectodermal cells into the midline lineage of the central nervous system (CNS). It is essential at all stages of their development—from precursor formation to differentiation into mature neurons and glia. Moreover, SIM regulates the transcription of all genes specific to these cells. Its ectopic expression can transform lateral neuroectodermal cells into CNS midline lineage cells (Franks and Crews, 1994; Coban et al., 2020; Yamaki et al., 2001). SIM2 is located within the critical region of Down syndrome in the human genome, suggesting that it may play a role in the complex etiology of this disorder. Additionally, studies conducted on mice overexpressing SIM2 have demonstrated deficits in learning abilities, indicating a potential impact of this protein on cognitive functions. Although current evidence suggests an association between SIM2 and Down syndrome, its precise role in the pathogenesis of this condition requires further detailed investigation (Chen et al., 1995; Ema et al., 1999; Chrast et al., 2000). SIM expression has also been identified in the kidneys and muscles; however, its functions in these tissues are not well characterized. SIM1 is critical in the neuronal circuit regulating appetite. SIM1-expressing neurons play a key role in maintaining the body’s energy homeostasis. Mutations in Sim1 cause obesity and hyperphagia in both rodents and humans (Nyamugenda et al., 2019; Holder et al., 2000) Previous studies using human and mouse models have indicated that SIM is also an important factor in cancers of the colon, prostate, pancreas, and breast (Deyoung et al., 2003; Aleman et al., 2005; Laffin et al., 2008; Arredouani et al., 2009; Wellberg et al., 2010).

**FIGURE 5:**
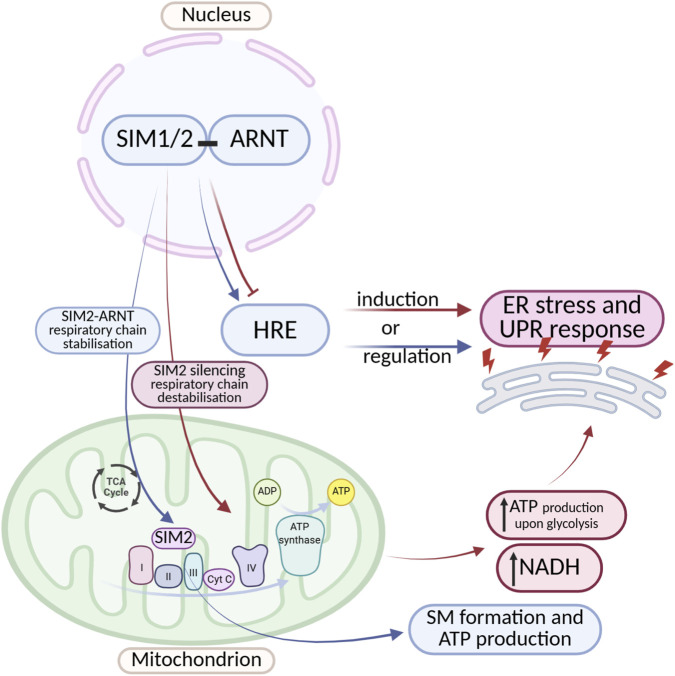
Crosstalk Between SIM Transcription Factors and ER Stress/UPR Signaling. A complex, bidirectional regulation exists between SIM proteins and ER stress as well as the UPR. SIM proteins can both induce and inhibit ER stress and UPR pathway activity. They act by modulating the expression of genes containing HREs, as well as by influencing the stability of the mitochondrial electron transport chain. Despite an increasing number of reports highlighting the important role of SIM proteins in regulating cellular stress responses, the detailed mechanisms underlying these interactions remain incompletely understood. The diagram was created based on the description provided in Section 2.2. (Figure prepared with BioRender).

To date, no studies have directly or indirectly examined the influence of SIM1/2 on ER stress. However, an indirect role of SIM proteins in ER stress regulation can be hypothesized, partly due to their ability to bind hypoxia response elements and modulate transcription. The dimerization partner of SIM1 and SIM2 is ARNT, and the resulting SIM1-ARNT and SIM2-ARNT complexes can interact with both E-box sequences and HREs, which are commonly the binding sites for HIF-1α. Acting as a transcriptional repressor, SIM2 may inhibit the expression of HIF-1α target genes, whereas the SIM1-ARNT dimer can facilitate their activation. Depending on the prevailing dimer configuration, the hypoxic response may be either potentiated or suppressed, thereby modulating the magnitude of ER stress induced by oxygen deprivation (Figure 5) (Woods and Whitelaw, 2002; Bersten et al., 2013).

Another interesting example of the impact of SIM proteins on ER stress is the disruption of cellular energy metabolism. Studies indicate that in breast cancer cells, SIM2 promotes mitochondrial oxidative phosphorylation (Wall et al., 2023) Wall et al. (2023) observed, that SIM can localize to mitochondria and directly interact with the electron transport chain, forming a protein supercomplex (S.C.) responsible for ATP production. Silencing of SIM2 leads to destabilization of Complex II of the electron transport chain. This contributes to inhibited electron transport, compensatory increases in ATP production via glycolysis, and elevated NADH production (driven by glutamine metabolism). This mechanism creates an environment that promotes cancer cell proliferation. These findings suggest that SIM2 supports proper electron transport chain function, and loss of its expression may disrupt electron transport, leading to excessive production of reactive oxygen species (ROS), which damage ER proteins and trigger ER stress and the UPR (Figure 5) (Bersten et al., 2013).

### 2.3 Aryl hydrocarbon receptor (AHR)

**FIGURE 6:**
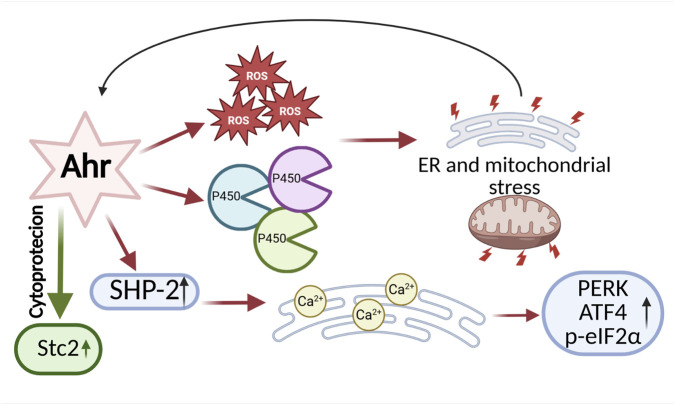
Bidirectional Effects of AHR on ER Stress and Adaptation. On one hand, AHR can promote ER stress by enhancing the production of ROS, activating cytochrome P450 enzymes, and stimulating the activity of the phosphatase SHP-2, which disrupts calcium (Ca^2+^) homeostasis. On the other hand, AHR can exert cytoprotective functions by supporting the expression of genes that regulate ER homeostasis, such as Stanniocalcin 2. This bidirectional activity highlights the complex role of AHR in balancing cellular adaptation and stress. The diagram was created based on the description provided in Section 2.3. (Figure prepared with BioRender).

The aryl hydrocarbon receptor (AHR) is the only transcription factor from the bHLH-PAS family for which activating ligands are known. Its primary function is the regulation of xenobiotic metabolism, including dioxins, which are exogenous ligands that activate AHR to initiate the detoxification pathway. In its inactive state, AHR resides in the cytoplasm as part of a complex with chaperone proteins such as HSP90 (Heat Shock Protein 90), the co-chaperone p23, and ARA9 (Aryl Hydrocarbon Receptor-Associated Protein 9). HSP90 stabilizes the receptor and supports its ligand-binding capacity while preventing premature translocation of AHR to the nucleus. Meanwhile, p23 plays a key role in stabilizing the AHR-HSP90 complex, maintaining its structural integrity (Pongratz et al., 1992; Carver et al., 1998). Upon ligand binding, AHR translocates to the nucleus, where it forms a heterodimer with ARNT (Aryl Hydrocarbon Receptor Nuclear Translocator, also known as HIF-1β). The AHR-ARNT complex then binds to XRE (Xenobiotic Response Elements) sequences in the promoters of target genes, leading to the regulation of their expression. Consequently, activation of AHR induces the expression of biotransformation enzymes belonging to the cytochrome P450 family, such as CYP1A1 and CYP1B1, which are involved in xenobiotic detoxification and the cellular response to environmental stress (Granados et al., 2022; Ye et al., 2019). There are also reports indicating that AHR activation can occur independently of exposure to xenobiotics, and that its induction may be promoted by endogenous, natural ligands. These studies have provided evidence for a physiological role of AHR in the regulation of cellular functions (Singh et al., 1996; Richterb et al., 2001; Kawajiri and Fujii-Kuriyama, 2017). AHR is involved in the modulation of immune and inflammatory processes, influencing, among others, the function of regulatory T cells (Tregs), Th17 lymphocytes, and dendritic cells. This modulation may play a significant role in immune homeostasis as well as in the pathogenesis of autoimmune diseases and cancers (Rothhammer and Quintana, 2019). Furthermore, AHR plays a significant role in the regulation of angiogenesis, exhibiting both pro-angiogenic and anti-angiogenic effects depending on the microenvironmental conditions and the endothelial cell type (Li et al., 2020). By influencing the expression of genes regulating the cell cycle, AHR can modulate cell proliferation, contributing to its role in tumorigenesis. Its activity may either promote tumor progression or exert antitumor effects, depending on the biological context and cancer type. (Yin et al., 2016; Xue et al., 2018). Similar to the transcription factors SIM1/2, information regarding the role of AHR in regulating ER stress is limited, and the available research findings remain inconclusive. It has been shown that AHR can both exacerbate and alleviate ER stress, depending on the experimental conditions and cell type. Mechanisms by which AHR contributes to increased ER stress include, among others, the induction of reactive ROS production and activation of cytochrome P450 family enzymes involved in biotransformation processes. Since these enzymes are localized in the ER membrane, their excessive expression or activity may lead to overload of the detoxification system and ER dysfunction, thereby contributing to an enhanced cellular stress response (Shimada et al., 2009; Zhang et al., 2022a). Treatment of JHP lymphoblast cells with one of the exogenous AHR ligands, hydroquinone (HQ), activates AHR and leads to excessive production of ROS, followed by activation of CYP1A1 transcription. Together with ROS, this promotes ER stress and the UPR (Figure 6) (Yang et al., 2023). The increased expression of cytochrome P450 enzymes suggests that AHR ligands promote ROS generation in microsomes of the endoplasmic reticulum and mitochondria, contributing to the activation of both ER stress and mitochondrial stress (Senft et al., 2002a; Senft et al., 2002b). Wang and colleagues demonstrated that the tyrosine phosphatase SHP-2 plays a key role in the activation of ER stress in response to AHR signaling. In studies conducted on mast cells, it was observed that both AHR stimulation by the ligand FICZ and SHP-2 activity significantly affect calcium homeostasis in the ER, which serves as the primary intracellular reservoir of Ca^2+^ ions (Figure 6). These processes resulted in ROS-dependent calcium release into the cytosol, leading to activation of the ER stress response through induction of PERK, ATF4 expression, and phosphorylation of eIF2α (Wang et al., 2017). Activation of ER stress via an AHR-dependent mechanism has also been observed in mouse hippocampal neurons. α-Naphthoflavone, an AHR activator, induces apoptotic cell death through activation of caspases 12 and 13, as well as increased expression of ER stress markers such as CHOP. Inhibition of AHR activity using the antagonist CH223191 and AHR knockdown reduced, among other effects, CHOP expression and cell death (Yu et al., 2019). In contrast, Guerrina et al. demonstrated that AHR can mitigate ER stress. In AHR knockout (AHR^−/−^) lung cells treated with cigarette smoke extract, they observed increased expression of ER stress markers CHOP and GADD34, as well as accumulation of ubiquitinated proteins (Guerrina et al., 2021). Studies conducted by Joshi et al. provided evidence for the cytoprotective role of the AHR pathway in the context of ER stress. Using isolated primary hepatocytes from wild-type and conditional AHR knockout mice, they demonstrated that AHR activation by citric acid directly upregulates the expression of 2 (Stc2). This expression plays a crucial role in protecting cells against apoptosis induced by ER stress (triggered by thapsigargin) and oxidative stress (induced by hydrogen peroxide - H_2_O_2_) (Joshi et al., 2015). Interestingly, studies also indicate that not only can AHR modulate ER stress, but ER stress itself can induce AHR expression. In in vivo studies using a mouse model of PCOS, it was observed that heightened ER stress promotes the upregulation of AHR, ARNT, as well as CYP1B1 (Kunitomi et al., 2021). Research on the role of AHR in the context of ER stress is still at an early stage. Existing findings indicate that AHR is involved in the regulation of ER stress and the UPR. However, the observed mechanisms of AHR action are varied, and the data obtained remain inconclusive, necessitating further studies to fully elucidate its function in this process (Figure 6).

### 2.4 Circadian locomoter output cycles protein kaput (CLOCK)

The circadian rhythm is a natural internal biological clock of the body. It regulates physiological processes such as body temperature, blood pressure, metabolism and digestion, sleep and wakefulness, immune system responses, and hormone secretion. Therefore, it plays a key role in maintaining the body’s homeostasis (Patke et al., 2020). The central circadian rhythm oscillator is the suprachiasmatic nucleus (SCN), located in the hypothalamus. It regulates the activity of peripheral oscillators found in various organs and tissues. At the cellular level, the function of oscillators is carried out by circadian clock genes. The core feedback loop of the biological clock is driven by transcription factors CLOCK, BMAL1 (brain and muscle ARNT-like 1), and NPAS2 (neuronal PAS domain protein 2), all of which belong to the bHLH-PAS family of transcription factors (Ko and Takahashi, 2008). At the cellular level, oscillators operate through positive and negative translational or post-translational feedback loops. The positive feedback loop mechanism is based on the heterodimerization of CLOCK and BMAL1, resulting in the formation of a complex that promotes the transcription of target genes containing E-box sequences, such as Period (Per1, Per2, Per3) and Cryptochrome (Cry1, Cry2). The PER:CRY complexes function via negative feedback. They form in the cytoplasm and then translocate to the nucleus, where they inhibit the activity of the CLOCK:BMAL1 complex, thereby blocking the transcription of genes, including their own—Per and Cry (Patke et al., 2020; Lee et al., 2001). An additional feedback loop supporting the function of the biological clock is also initiated by the CLOCK:BMAL1 complex, which activates the transcription of the nuclear receptors REV-ERBα and RORα. These receptors compete for binding to RORE (ROR response elements) within the promoter region of the BMAL1 gene. REV-ERBα acts as a transcriptional repressor, inhibiting BMAL1 expression, whereas RORα functions as a transcriptional activator, promoting its expression. The antagonistic interaction between these factors contributes to the precise regulation and stabilization of the circadian rhythm (Akashi and Takumi, 2005; Guillaumond et al., 2005).

**FIGURE 7:**
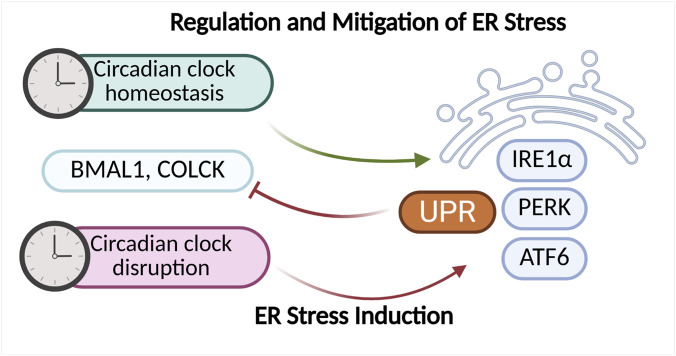
Factors regulating the circadian rhythm and mechanisms responsible for ER homeostasis exhibit mutual interactions. Proper functioning of the molecular clock supports the maintenance of balance within the endoplasmic reticulum, whereas its disruption can lead to the activation of ER stress and induction of the UPR response. Conversely, chronic or intensified ER stress can negatively affect the expression of key molecular clock components, such as BMAL1 and CLOCK, further exacerbating disturbances in the circadian rhythm and cellular homeostasis. The diagram was created based on the description provided in Section 2.4. (Figure prepared with BioRender).

Existing research findings indicate that the circadian rhythm, and especially the oscillators functioning at the cellular level, play a significant role in regulating ER stress and the UPR response. There is also evidence suggesting a reciprocal relationship between these mechanisms. One of the main UPR pathways modulated by the circadian rhythm is the IRE1α–XBP1 pathway. The UPR plays a key role in regulating hepatic lipid metabolism, which is also subject to circadian rhythmicity. Studies using a mouse liver model exposed to ER stress-inducing agents demonstrated that activation of the IRE1α–XBP1 pathway occurs in a 12-h rhythmic pattern. Moreover, mice lacking a functional biological clock exhibited impaired UPR activation, leading to deregulation of enzymes localized in the endoplasmic reticulum and consequently to disturbances in lipid metabolism (Cretenet et al., 2010; Lee and Glimcher, 2009). Other studies have revealed that UPR activation via induction of miR-211 (a PERK-induced microRNA) leads to 10-h shifts in circadian oscillations. These shifts result from the transient repression of Bmal1 and Clock activity. miR-211 regulates Bmal1 and Clock through distinct mechanisms. It was also observed that repression of Bmal1 is essential for UPR-dependent inhibition of protein synthesis and cellular adaptation to ER stress. These findings suggest that Bmal1 suppression via UPR activation in Burkitt lymphoma dampens circadian oscillations and protein synthesis, thereby promoting tumor progression (Bu et al., 2018). Disruptions of the circadian rhythm can lead to activation of ER stress and initiation of the UPR response (Figure 7). Simultaneously, dysfunctions in ER can disturb the homeostasis of the biological clock, leading to its dysregulation. The reciprocal relationship between these mechanisms may contribute to the development of numerous metabolic diseases as well as cancers. Lee et al. (2016) demonstrated that both genetic and environmental disruptions of the molecular clock—mainly through impaired transcriptional regulation of Clock and Bmal1—lead to oxidative stress and ER stress in pancreatic β-cells. These disturbances are accompanied by mitochondrial dysfunction and β-cell failure (Lee et al., 2015). Restoration of molecular clock homeostasis may alleviate ER stress. In a mouse model subjected to light deprivation, circadian rhythm disruption led to decreased expression of the Bmal1 protein in the liver and activation of the ER stress pathway, manifested by increased expression of markers such as GRP78, ATF4, and CHOP (Figure 7). Administration of a low dose of compound SX (SXL) restored normal circadian activity rhythms, metabolic patterns, and reduced liver damage and ER stress in mice with disrupted circadian rhythms (Zhang et al., 2022b). The latest study by Erzurumlu et al. (2024) revealed a daily oscillation pattern of UPR signaling in a circadian rhythm in human kidney HEK293 cells. UPR response markers such as IRE1A, XBP1, eIF2α, phospho (Ser51)-eIF2α, PERK, ATF4, GADD34, and ATF4 exhibited a 48-h oscillatory pattern. This observed pattern was similar to the oscillation of the PER1 gene, a core component of the circadian clock (Erzurumlu et al., 2024). These findings represent a significant step toward the development of UPR-targeted therapies that take physiological circadian regulation into account, potentially enhancing their efficacy and safety. Studies using the *Litopenaeus vannamei* model demonstrated that the expression of circadian rhythm markers depends on the UPR response, with particular importance attributed to the PERK-eIF2α pathway. It was observed that UPR induction by tunicamycin led to changes in the expression of circadian genes such as LvCry1, LvPeriod, LvTimeless, and LvVrille. Phase shifts and reduced amplitude of these genes’ expression were noted, suggesting disruption of the circadian rhythm alongside effects on metabolic and immune processes. There is also evidence indicating that disturbances in ER stress signaling affect the biological clock and the transcription of its regulated genes. In studies using a mouse NIH3T3 fibroblast model treated with ER stress activators (thapsigargin or tunicamycin), a significant reduction in the amplitude of Bmal1 oscillations and phase delay shifts were observed. ER stress activators induced stress through upregulation of GRP78, CHOP, ATF6, and ATF4 expression, while simultaneously reducing BMAL1 protein levels (Figure 7). Additionally, transcription of clock genes (Bmal1, Per2, Nr1d1, and Dbp) and clock-controlled genes (Scad1, Fgf7, and Arnt) was suppressed. Treatment with the ER stress inhibitor 4-phenylbutyric acid (4PBA) alleviated transcriptional repression and partially restored normal Bmal1 oscillation amplitudes. These results also suggest that the mechanism by which ER stress inhibits the biological clock is primarily dependent on ATF4 (Gao et al., 2019). Recent studies by Gao et al. also demonstrated the impact of ER stress on the biological clock. It was observed that in aged animals (mice), age-associated ER stress inhibits testosterone synthesis by suppressing the biological clock in Leydig cells (Gao et al., 2022). The circadian rhythm and molecular clock play a crucial role in regulating ER stress and the UPR response, with disruptions in these mechanisms mutually exacerbating each other. This interplay leads, among other effects, to dysregulation of lipid metabolism, oxidative stress, as well as mitochondrial dysfunction and impaired protein synthesis. ER stress can suppress the activity of key circadian genes (e.g., Bmal1, Clock), while restoration of molecular clock balance reduces ER stress, opening new therapeutic perspectives (Figure 7).

### 2.5 Neuronal PAS domain protein (NPAS)

Among the proteins belonging to the bHLH-PAS family, the least studied group in the context of ER stress are those from the NPAS proteins subfamily, which includes NPAS1, NPAS2, NPAS3, and NPAS4. Of these, NPAS2 is the best characterized, with expression demonstrated primarily in the central nervous system—where it reaches its highest levels—as well as in endocrine tissues, the respiratory system, the female reproductive system, and cardiac muscle. NPAS2 functions as a functional analog of the CLOCK factor, forming heterodimers with BMAL1 and participating in the regulation of circadian rhythms (Reick et al., 2001). Studies using a mouse model with a knockout of the Clock gene suggest that NPAS2 is capable of functionally compensating for CLOCK in maintaining the homeostasis of the biological clock (DeBruyne et al., 2007). Increasing evidence suggests that NPAS2 may also play a role in regulating cellular metabolism and the response to oxidative stress by modulating the expression of genes responsible for the production of ROS and antioxidants (Rutter et al., 2001; Becker-Krail et al., 2022; O’Neil et al., 2013). However, definitive evidence confirming the direct involvement of NPAS2 in regulating ER stress and activating the UPR pathway is still lacking. There are only indications suggesting that the heterodimeric complexes CLOCK (or NPAS2):BMAL1 may regulate the expression of clock genes that influence ROS production and thereby indirectly modulate ER stress (McClung, 2013).

The transcription factors NPAS1 and NPAS3 play important roles in regulating the function of the central nervous system. Their expression is particularly prominent in the brain — NPAS1 is present in regions such as the hippocampus, amygdala, and cerebral cortex, while NPAS3 is found in the hippocampus, basal ganglia, cerebral cortex, amygdala, and midbrain. Dysregulation or genetic deficiencies of these proteins have been linked to various neuropsychiatric disorders, including schizophrenia, autism, and bipolar disorder (Kamnasaran et al., 2003; Stanco et al., 2014; Pickard et al., 2009). For transcriptional activity, both NPAS1 and NPAS3 must form heterodimers with the ARNT. Despite sharing the same dimerization partner, these proteins perform distinct functions — NPAS1 primarily acts as a transcriptional repressor, whereas NPAS3 functions as a transcriptional activator (Teh et al., 2006; Luoma and Berry, 2018).

There is only limited evidence suggesting a potential indirect involvement of NPAS1 and NPAS3 proteins in the regulation of ER stress and the UPR. Current data indicate that in schizophrenia, NPAS3 may aggregate in the insular cortex, associated with the presence of the V304I point mutation. Importantly, in a neuroblastoma cell model, it was shown that NPAS3 aggregation is enhanced under oxidative stress, which may suggest a link between disrupted redox homeostasis and conformational changes of the NPAS3 protein. Although this connection has not yet been directly associated with ER stress or UPR activation, these observations highlight the potential significance of NPAS3 in the cellular stress response and warrant further investigation (Samardžija et al., 2021).

NPAS4 expression is most prominent in the central nervous system, particularly in structures such as the cerebral cortex, hippocampus, basal ganglia, and cerebellum. For a long time, the expression of this transcription factor was believed to be limited exclusively to the brain. However, more recent data, including analyses from the Human Protein Atlas database, indicate its low-level expression in other tissues as well, such as endocrine tissues, the gastrointestinal tract (e.g., colon, stomach), female and male reproductive tissues (e.g., endometrium, prostate), as well as the pancreas and kidneys. Most of the existing research has focused on the function of NPAS4 in the brain, where it plays a key role in regulating the expression of genes associated with neuroplasticity, neuroprotection, and processes involved in memory and learning (Weng et al., 2018; Yun et al., 2013; Choy et al., 2015; Sun and Lin, 2016). Therefore, it is considered a promising therapeutic target in the context of neurodegenerative diseases, including Alzheimer’s disease (Fan et al., 2016). Moreover, there are reports suggesting the involvement of NPAS4 in the regulation of endothelial cell functions and in the process of angiogenesis, that is, the formation of new blood vessels (Esser et al., 2017; Kobayashi et al., 2020).

It is also worth emphasizing that NPAS4 belongs to the immediate early genes (IEGs), which activate mechanisms associated with the first line of defense under cellular stress conditions (Sun and Lin, 2016). Their unique importance lies in the fact that they represent the first wave of transcriptional response induced by diverse external stimuli, including stress factors. The expression of these genes occurs very rapidly—usually within minutes of stimulus onset—and importantly, does not require prior protein synthesis because their promoters are activated by pre-existing, already activated signaling pathways in the cell (e.g., MAPK, ERK, JNK, p38). The protein products of immediate early genes are most often transcription factors that initiate a second wave of target gene expression responsible for adapting the cell to altered conditions. This includes regulation of proliferation, differentiation, synaptic plasticity, immune response, as well as protective pathways against apoptosis and oxidative stress. In the context of cellular stress, IEGs thus function as sensors and regulators of the early response, reprogramming the cell’s transcriptome in a way that enables either survival or activation of programmed cell death, depending on the nature and intensity of the stimulus (Bahrami and Drabløs, 2016).

**FIGURE 8:**
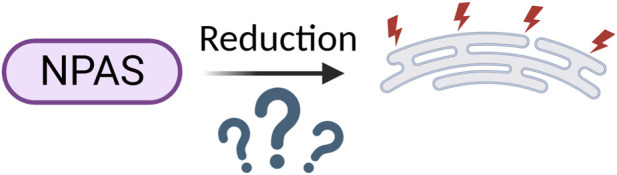
The Role of Neuronal PAS Domain Proteins in Modulating ER Stress: Current Insights and Gaps in Understanding Mechanisms. (Figure prepared with BioRender).

Scientific literature also reports suggest potential interactions between the transcription factor NPAS4 and ER stress, which could partly explain its neuroprotective and cytoprotective properties, the mechanisms of which remain incompletely understood. Studies by Sabatini et al., using mouse MIN6 β-cell lines as well as isolated mouse and human pancreatic islets, demonstrated that the expression of the Npas4 gene is significantly upregulated not only in response to β-cell depolarizing stimuli but also following exposure to classical ER stress inducers such as thapsigargin and palmitate. Furthermore, it was observed that NPAS4, through direct interaction with the insulin gene promoter, inhibits the cellular response to glucagon-like peptide 1 (GLP-1), thereby reducing insulin levels. Based on these findings, the authors concluded that Npas4 may represent a novel therapeutic target for diabetes treatment and functions as an early mediator of the cellular stress response, potentially protecting cells from ER stress overload and supporting the maintenance of their functional homeostasis (Sabatini et al., 2013). In other studies, Sabatini et al. observed that NPAS4 functions as a negative regulator of the hypoxia response in neuroendocrine cells. Under hypoxic conditions (1% O_2_), NPAS4 inhibits the expression of Hif-1α and limits the formation of the HIF-1α/ARNT complex, resulting in the suppression of transcription of hypoxia-responsive genes such as Vegfa, Ldha, and Pdk1 (Sabatini et al., 2018) In this way, NPAS4 exerts a protective effect by reducing excessive metabolic activation and the resulting overload of the ER. Although a direct link between NPAS4 and UPR pathways has not yet been fully described, its activity suggests a role in mitigating ER stress through suppression of the HIF-1α axis and stabilization of oxygen metabolism (Figure 8).

## 3 Summary

Proteins from the bHLH-PAS family are central regulators of cellular adaptation. They integrate diverse environmental, metabolic, and stress-related signals to orchestrate gene expression programs that are essential for maintaining homeostasis. In recent years, increasing evidence has highlighted their potential involvement in the regulation of ER stress and the UPR. However, the scope and nature of this interaction remain incompletely defined and highly context-dependent. There is also a limited but growing body of evidence indicating that modulators of bHLH-PAS family proteins — including both agonists and antagonists — can influence endoplasmic reticulum (ER) stress (Tabe 1).

**TABLE 1:** Examples of pharmacological modulators of AHR, HIF, SIM, NPAS factors and circadian clock components, and their impact on ER stress.

Protein	Modulator type	Examples of molecules	Reported impact on ER stress/UPR
AHR	Agonists	TCDD (Duan et al., 2014) Benzo(a)pyrene (Siangcham et al., 2025)	↑ER stress via ROS generation, inflammation, lipid dysregulation (Duan et al., 2014) ↑ER stress via BiP, PERK and IRE1 (Siangcham et al., 2025)
	Antagonist	CH-223191 (Guerrina et al., 2021) Resveratrol (Lee et al., 2019) α-Naphthoflavone (Yu et al., 2019)	↑ER stress markers (CHOP, GADD34) (Guerrina et al., 2021) ↓ER stress (↓ p-PERK, p-IRE1α, CHOP, ↑ERAD factors: SEL1L, HRD1) (Lee et al., 2019) ↑ER stress by ↑CHOP, MPAK and ROS (Yu et al., 2019)
HIF-1/2	Stabilizers	DMOG (Han et al., 2025); Daprodustat (Tóth et al., 2024)	↓ER stress by inhibiting ATF6, GRP78/BiP i CHOP) (Han et al., 2025) ↑ER by ATF4 indution (Tóth et al., 2024)
	Inhibitors	Acriflavine (Martí-Díaz et al., 2021; El-Waseif et al., 2025) PX-478 (Zhao et al., 2015)	↑ER stress by ↑eIF2α and ATF4 destabilisation (Martí-Díaz et al., 2021) ↓ER stress by inhibition PERK/PI3K) (El-Waseif et al., 2025) PX-478 directly provoked ER stress but ↓ eIF2α (Zhao et al., 2015)
SIM1/SIM2	No well-characterized agonists or antagonists reported		
NPAS1–4	No well-characterized agonists or antagonists reported		
CLOCK	Circadian modulators	SR9009 (REV-ERB agonist) (Wang et al., 2023) PF-670462 (CK1δ/ε inhibitor) (Hicks et al., 2020)	↓ER stress by ↓GRP78 and ↓autophagy (Wang et al., 2023) ↓ER stress by ↓phosphorylation of CK1δ/ε substrates that contribute to protein aggregation in ER (Hicks et al., 2020)

**FIGURE 9:**
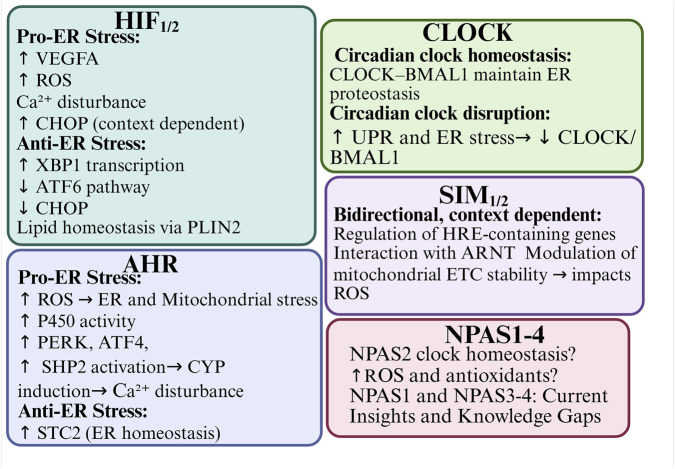
Integrated graphical abstract of bHLH-PAS transcription factors in the context of ER stress.

Transcription factors, including HIF-1α, HIF-2α, AHR, SIM1/2, and NPAS4,have been shown to modulate ER stress directly or indirectly. This modulation occurs via mechanisms such as ROS generation, calcium signaling disruption, and the regulation of UPR-related genes (e.g., *CHOP*, *ATF4*, *XBP1*). For example, HIF-1α and HIF-2α are activated under hypoxic conditions and can either alleviate or promote ER stress, depending on the duration and intensity of oxygen deprivation. Similarly, AHR can exacerbate ER stress through cytochrome P450 enzyme activity in response to xenobiotic ligands. Alternatively, AHR can act cytoprotectively by modulating apoptosis-related pathways. SIM proteins may influence ER stress by competing with HIF-1α for ARNT binding during transcription. NPAS4 appears to function as an early responder to ER stress and hypoxia, limiting metabolic overload and protecting against ER dysfunction. It is notable that bHLH-PAS proteins are deeply interconnected with circadian rhythm regulation, particularly through CLOCK, BMAL1 and NPAS2. These proteins control cellular oscillators and are themselves sensitive to ER stress-induced feedback. Disruption of the circadian clock leads to exacerbated ER stress. Conversely, restoration of clock gene activity has been alleviates stress responses, indicating a mutual regulatory loop Figure 9. An interesting aspect that needs further research is heme binding by NPAS2 and CLOCK. Binding of signaling gases like carbon monoxide (CO) and nitric oxide (NO) enables signal transduction and possible mechanism of regulatory control. CO acting as putative ER stress attenuator by reducing C-reactive protein is produced by degradation of heme by the O_2_-dependent heme oxygenase enzyme, and NO - the O_2_-dependent NO synthase enzyme product, that was shown as protective during ER stress in pancreatic β-cells (Riccio et al., 2006; Shimizu et al., 2019). Furthermore, chronic ER stress can alter the expression and stability of bHLH-PAS proteins, which further reinforces the bidirectional nature of this relationship.

Despite growing insights, the direct mechanistic links between many bHLH-PAS proteins and specific branches of the UPR (PERK, IRE1α, and ATF6) are still not well understood. Most findings are limited to specific cell types or disease models such as cancer, neurodegeneration, and metabolic disorders. Overall, the interplay between bHLH-PAS transcription factors and ER stress reflects a highly dynamic and multilayered regulatory network. Understanding this relationship has promising therapeutic potential for diseases involving disturbed proteostasis, oxidative damage, or impaired stress responses. Further comprehensive research is essential to unravel the precise molecular mechanisms and identify viable intervention points within this system.
